# Very high prevalence of extended-spectrum beta-lactamase-producing *Enterobacteriaceae* in bacteriemic patients hospitalized in teaching hospitals in Bamako, Mali

**DOI:** 10.1371/journal.pone.0172652

**Published:** 2017-02-28

**Authors:** Samba Adama Sangare, Emilie Rondinaud, Naouale Maataoui, Almoustapha Issiaka Maiga, Ibrehima Guindo, Aminata Maiga, Namory Camara, Oumar Agaly Dicko, Sounkalo Dao, Souleymane Diallo, Flabou Bougoudogo, Antoine Andremont, Ibrahim Izetiegouma Maiga, Laurence Armand-Lefevre

**Affiliations:** 1 Bacteriology Laboratory, Centre Hospitalier Universitaire Gabriel Touré, Bamako, Mali; 2 Bacteriology Laboratory, Centre Hospitalier Universitaire Bichat-Claude Bernard, Paris, France; 3 Faculté de Pharmacie, Université des Sciences, des Techniques et des Technologies de Bamako, Bamako, Mali; 4 INSERM, IAME, UMR 1137, Université Paris Diderot, Sorbonne Paris Cité, Paris, France; 5 Institut National de Recherche en Santé Publique, Bamako, Mali; 6 Bacteriology Laboratory, Centre Hospitalier Universitaire du Point G, Bamako, Mali; 7 Faculté de Médecine et d’Odonto- stomatologie, Université des Sciences, des Techniques et des Technologies de Bamako, Bamako, Mali; 8 Centre d’Infectiologie Charles Mérieux, Bamako, Mali; Universidad de Santiago de Compostela, SPAIN

## Abstract

The worldwide dissemination of extended-spectrum beta-lactamase producing *Enterobacteriaceae*, (ESBL-E) and their subset producing carbapenemases (CPE), is alarming. Limited data on the prevalence of such strains in infections from patients from Sub-Saharan Africa are currently available. We determined, here, the prevalence of ESBL-E/CPE in bacteriemic patients in two teaching hospitals from Bamako (Mali), which are at the top of the health care pyramid in the country. During one year, all *Enterobacteriaceae* isolated from bloodstream infections (E-BSI), were collected from patients hospitalized at the Point G University Teaching Hospital and the pediatric units of Gabriel Touré University Teaching Hospital. Antibiotic susceptibility testing, enzyme characterization and strain relatedness were determined. A total of 77 patients had an E-BSI and as many as 48 (62.3%) were infected with an ESBL-E. ESBL-E BSI were associated with a previous hospitalization (OR 3.97 95% IC [1.32; 13.21]) and were more frequent in hospital-acquired episodes (OR 3.66 95% IC [1.07; 13.38]). Among the 82 isolated *Enterobacteriaceae*, 58.5% were ESBL-E (20/31 *Escherichia coli*, 20/26 *Klebsiella pneumoniae* and 8/15 *Enterobacter cloacae*). The remaining (5 *Salmonella* Enteritidis, 3 *Morganella morganii* 1 *Proteus mirabilis* and 1 *Leclercia adecarboxylata)* were ESBL negative. CTX-M-1 group enzymes were highly prevalent (89.6%) among ESBLs; the remaining ones being SHV. One *E*. *coli* produced an OXA-181 carbapenemase, which is the first CPE described in Mali. The analysis of ESBL-E relatedness suggested a high rate of cross transmission between patients. In conclusion, even if CPE are still rare for the moment, the high rate of ESBL-BSI and frequent cross transmission probably impose a high medical and economic burden to Malian hospitals.

## Introduction

Bacterial resistance to antibiotics is increasing worldwide in healthcare settings and in the community. The dissemination of multi-drug resistant *Enterobacteriaceae*, *i*.*e*. extended spectrum beta-lactamase (ESBL-E) and carbapenemase producing *Enterobacteriaceae* (CPE), is alarming [1]. Because of the global spread of CTX-M enzyme, ESBL-E are the cause of increasing numbers of both nosocomial and community infections [2]. Consequently, the use of carbapenems, which are the first choice drug to treat ESBL-E severe infections, has increased. Thus, carbapenem resistant bacteria, *i*.*e*. CPE, have emerged and disseminated all over the world, leaving few therapeutic options [3]. Severe infections due to multidrug resistant *Enterobacteriaceae* are associated with worst outcomes and increased mortality, especially when adequate antibiotic therapy is delayed [4–5]. Low and middle income countries (LMICs) are—and will be—particularly affected [6] but precise data from their hospitals are often lacking, preventing to implement proper control strategies. In addition infections are most often under-documented microbiologically and most antibiotic regimens are chosen empirically. This is for instance the case in Mali, belonging to the 30 poorest countries in the world (http://data.worldbank.org/). There, during one year, we prospectively investigated and characterized the prevalence of ESBL-E and CPE in bacteremic patients hospitalized at the University Teaching Hospitals of Bamako, which are at the top of the health care pyramid in the country. The results show that the prevalence of resistance is very high and cross transmission of resistant strains is rather frequent.

## Materials and methods

### Study design and definitions

This prospective study has been conducted from January 1 to December 31 2014, at the Point G University Teaching Hospital (PGUH) (550 adult beds) and in the pediatric department of Gabriel Touré University Teaching Hospital (GTUH) (110 beds), both located in Bamako, Mali. Blood cultures were drawn from patients with a temperature ≥ 39°C and suspect of invasive bacterial infection.

All patients with at least one blood culture positive containing an *Enterobacteriaceae* were considered as having an *Enterobacteriaceae* bloodstream infection (E-BSI) and were therefore included in the analysis.

Demographic data and comorbidities were prospectively recorded, as well as the history of previous hospitalization at any hospital during the previous year. Community acquired BSI episodes were defined by a positive blood culture drawn less than two days after admission into one of the two hospitals, while the hospital-acquired BSI were defined by a positive blood culture drawn more than two days after the admission. However, BSI episodes from patients with positive blood culture drawn within two days of admission and who had been hospitalized elsewhere within the two previous weeks were classified as “not determined”.

### Statistics

To determine the risks factors associated with ESBL-E BSI, continuous and categorical variables were compared using Student t-test and Chi square analysis.

### Strain analysis

For each blood culture, venous blood (8 to 10 mL from adults and 1 to 5 mL from children) was collected and injected directly into a BD Bactec Plus Aérobie/F (adults) or BD Bactec Peds Plus/F (children) blood culture bottle, which was then introduced and incubated into a Bactec 9050 (Becton Dickinson, Franklin Lakes, USA).

Positive blood cultures were plated on blood agar plates (bioMérieux, Marcy l'Etoile, France) and on Drigalski agar plates (bioMérieux, Marcy l'Etoile, France) if Gram negative bacilli were present on direct smear examination.

Strains isolated from blood culture were identified locally using the automated system VITEK^®^2 and/or the API 20E system (bioMérieux, Marcy l'Etoile, France). All *Enterobacteriaceae* were stored at -80°C and shipped to Bichat laboratory (Paris, France), where all identifications were confirmed using mass spectrometry MALDI-TOF (Bruker Daltonics, Wisenheim, France). Antimicrobial susceptibility and ESBL phenotype were determined by disk diffusion and interpreted according to EUCAST (www.eucast.org). Intermediate susceptibility results were considered as resistant. MICs were determined using E-test strips (bioMérieux, Marcy l’Etoile, France)

DNA was extracted with MagNA Pure-DNA Isolation and Purification (Kit III) using a MagNA Pure LC 2.0 Instrument (Roche Lifescience, Penzberg, Germany). ESBL positive strains were first PCR screened for *bla*_CTX-M-1 group,_ *bla*_CTX-M-9 group_ *and bla*_CTX-M-8-25 group,_ as described [7], and if negative for *bla*_TEM_, *bla*_SHV_ *bla*_GES_, *bla*_VEB_, and *bla*_PER,_ as described [8]. A CarbaR^®^ Test (GeneXpert^®^ Cepheid, USA) and a PCR of *bla*_OXA-48 like_ were performed on strains suspected to be CPE, as described [9].

Amplification products were sequenced by an automated sequencer ABI 3130 (Applied Biosystems, Les Ulis, France) and analyzed in GenBank (www.ncbi.nlm.nih.gov/blast/).

Genetic relatedness among ESBL producing *Escherichia coli*, *Klebsiella pneumoniae* and *Enterobacter cloacae*, was determined using the semi-automated repetitive-sequence-based PCR (rep-PCR) DiversiLab system (bioMérieux, Marcy-l’Etoile, France) according to the manufacturer’s recommendations. The results were then analyzed as described [10]. Strains with 95% similarity or more were considered as indistinguishable.

### Ethical considerations

This study was approved by the ethics committee of the Faculty of Pharmacy, Medicine and Odonto-stomatology, University of Sciences, Techniques and Technologies of Bamako (USTTB). A written consent was obtained from all adults and from the next of kin, caretakers, or guardians on behalf of the children included in this study.

## Results

### Patients

**Fig 1:**
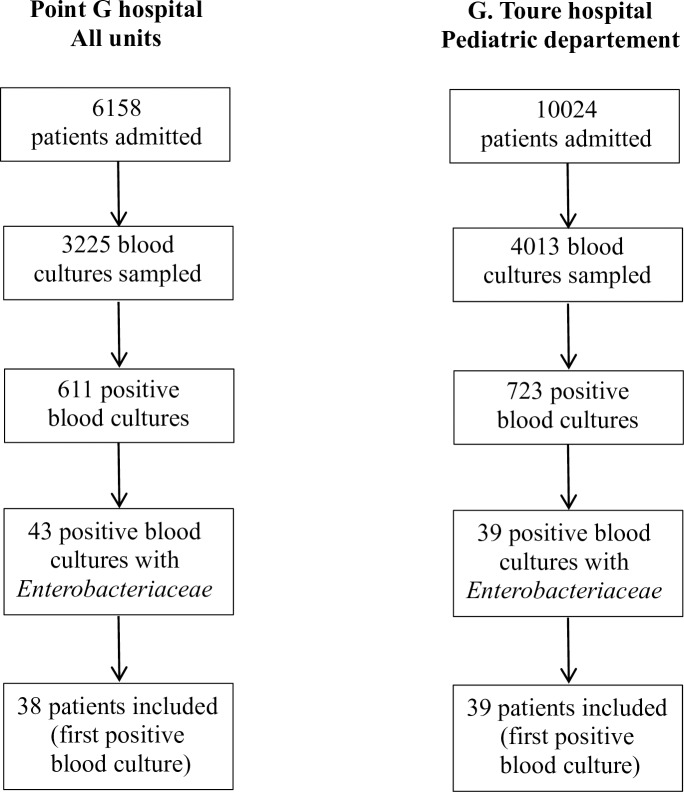
Flow diagram of patient selection.

During the study period, 6158 adults (PGUH) and 10024 children (GTUH) were admitted respectively to PGUH and GTUH. Overall, 3,225 and 4,013 blood cultures were collected respectively in each hospital. Among the 611 (18.9%) and 723 (18.0%) positive blood cultures, 43 and 39 contained an *Enterobacteriaceae* (E-BSI) in adults (PGUH) and children (GTUH), respectively. In all, 77 patients (38 adults and 39 children) had an E-BSI and were included in the study (Fig 1).

Patient’s characteristics are described in Table 1. Most episodes (70.1%) were hospital-acquired BSI, while 23.4% were community-acquired and 6.5% were not determined. In hospital-acquired BSI, interval days between admission and drawing of the first positive blood culture amounted to 28.5 in adults and 15.0 in children. In all, 48 episodes (62.3%) were ESBL positive (Table 1) with incidences of 3.90 and 2.39 per 1000 admissions in adults and children, respectively. ESBL rates in hospital-acquired BSI amounted to 67.9% (19/28) at PGUH and 73.1% (19/26) at GTUH. Previous hospitalization (OR 3.97 95% IC [1.32; 13.21]) and hospital-acquired episodes (OR 3.66 95% IC [1.07; 13.38]) were significant risks factors for ESBL-E BSI (Table 2).

**Table 1:** Characteristics of the patients.

	Point G UHosp	G. Touré UHosp	Total
Characteristics	.n = 38 (%)	.n = 39 (%)	n = 77 (%)
**Age, mean year (range)**	41.4 (15–80)	3.0 (0.4–13)	
**Male sex**	20 (52.6)	24 (61.5)	44 (57.1)
**Comorbidity**^**a**^	17 (44.7)	4 (10.3)	21 (27.3)
**Hospitalization within previous year**	19 (50)	14 (36.8)	33 (42.8)
**Hospital acquired episodes**	28 (73.7)	26 (66.6)	54 (70.1)
**Days between admission and bacteriemia, median**^**b**^ **(range)**	26 (1–97)	14 (1–43)	
**ESBL-E BSI**	24 (63.2)	24 (61.5)	48 (62.3)

^a^ Renal, cardiac or hepatic failure, cancer, HIV.

^b^ For hospitalized patients only.

**Table 2:** Risks factor for ESBL producing *Enterobacteriaceae* bloodstream infections.

Characteristics	n (%)	OR (95% IC)	P value
**Hospital**		-	0.93
Point G Hospital	24 (63.2)		
Gabriel Touré Ped. Dept.	24 (61.5)		
**Sex**		-	0.14
Male	31 (70.5)		
Female	17 (51.5)		
**Comorbidity**^**a**^		-	0.15
Yes	22 (73.3)		
No	26 (55.3)		
**Hospitalization within previous year**		3.97 (1.32–13.21)	0.009
Yes	27 (79.4)		
No	21 (48.9)		
**Origin of the BSI**^**b**^		3.66 (1.07–13.38)	0.03
Hospital-acquired	38 (70.4)		
Community-acquired	7 (38.9)		

^a^ Renal, cardiac or hepatic failure, cancer, HIV.

^b^ For 3 patients, the origin of the BSI was not determined.

### Microbiology

Among the 77 BSI episodes, 72 were monomicrobial and 5 contained two different *Enterobacteriaceae*. Thus, 82 *Enterobacteriaceae* were studied including 31 *E*. *coli* (37.8%), 26 *K*. *pneumoniae*, (31.7%), 15 *E*. *cloacae* (18.3%), 5 *Salmonella enterica* serotype Enteritidis (6.1%), 3 *Morganella morganii* (3.6%), 1 (1.2%) *Proteus mirabilis* and 1 (1.2%) *Leclercia adecarboxylata* (Table 3).

**Table 3:** Antibiotic resistance rate of *Enterobacteriaceae* isolated from bloodstream infections.

		Antibiotics											
Species	n	AMX	AMC	TZP	CTX	FEP	ERP	IMI	GE	AK	SXT	OFX	TE
		n (%)	n (%)	n (%)	n (%)	n (%)	n (%)	n (%)	n (%)	n (%)	n (%)	n (%)	n (%)
*Escherichia coli*	31	30 (96.8)	25 (80.6)	3 (9.7)	22 (71.0)^*b*^	20 (64.5)^*b*^	1 (3.2)^e^	0 (0)	17 (54.8)	0 (0)	24 (77.4)	24 (77.4)	29 (93.5)
*Klebsiella pneumoniae*	26	26 (100)	21 (80.8)	5 (19.2)	20 (76.9)^*c*^	20 (76.9)^*c*^	0 (0)	0 (0)	19 (73.1)	2 (7.7)	23 (88.5)	10 (38.5)	14 (53.8)
*Enterobacter cloacae*	15	15 (100)	15 (100)	1 (6.7)	9 (60.0)^*d*^	9 (60.0)^*d*^	0 (0)	0 (0)	7 (46.7)	0 (0)	8 (53.3)	8 (53.3)	8 (53.3)
*Other isolates* ^*a*^	10	10 (100)	7 (70.0)	0 (0)	0 (0)	0 (0)	0 (0)	0 (0)	0 (0)	0 (0)	7 (70.0)	2 (20.0)	9 (90.0)
**Total**	**82**	**81 (98.8)**	**68 (83.0)**	**9 (11.0)**	**51 (62.2)**	**49 (59.8)**	**1 (1.2)**	**0 (0)**	**43 (52.4)**	**2 (2.4)**	**62 (75.6)**	**44 (53.7)**	**60 (73.2)**

AMX: amoxicillin, AMC: amoxicillin + clavulanic acid, TZP: piperacillin + tazobactam, CTX: cefotaxime, FEP: cefepime, ERP: ertapenem, IMI: imipenem, GE: gentamicin, AK: amikacin, SXT: trimethoprim + sulfamethoxazol, OFX: ofloxacin, TE: tetracycline

^*a*^ Other isolates are *Salmonella* Enteritidis (n = 5), *Morganella morganii* (n = 3), *Proteus mirabilis* (n = 1) and *Leclercia adecarboxylata* (n = 1).

^*b*^ ESBL-*E*. *coli* n = 20

^*c*^ ESBL-*K*. *pneumoniae* n = 20

^*d*^ ESBL-E. *cloacae* n = 8

^e^ Carbapenemase producing *E*. *coli* n = 1.

**Fig 2:**
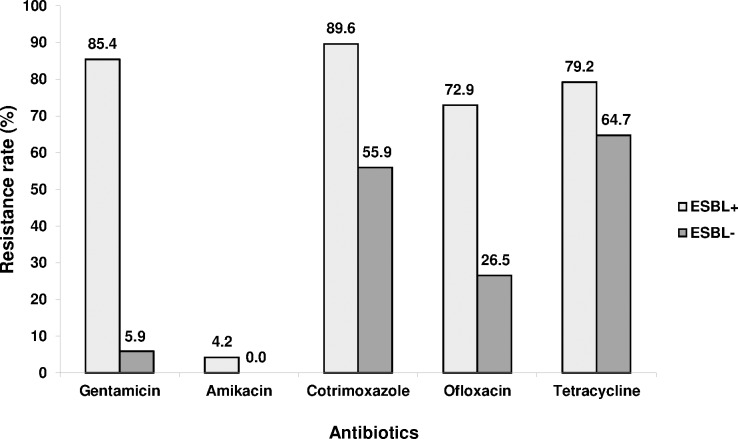
Antibiotic resistance rate according to the ESBL phenotype.

Resistance rates were 98.8% for amoxicillin, 75.6% for cotrimoxazole, 62.2% for third generation cephalosporin (48 ESBL and 3 non ESBL), 61.0% for amoxicillin + clavulanic acid, 53.7% for fluroquinolones, 52.4% for gentamicin, 11.0% for piperacillin + tazobactam, 2.4% for amikacin, and 1.2% for carbapenem (Table 3). The rate of ESBL producing strains were 76.9% (20/26) in *K*. *pneumoniae*, 64.5% (20/31) in *E*. *coli* and 53.3% (8/15) in *E*. *cloacae*. One *E*. *coli* produced an ESBL and a carbapenemase (Table 4). The co-associated antibiotic resistance rates were higher in ESBL-E than in non ESBL ones, 89.6 and 55.9% for cotrimoxazole, 85.4 and 5.9% for gentamicin, 72.9 and 26.5% for fluoroquinolones, respectively (p<0.05) (Fig 2).

**Table 4:** Prevalence and characterization of ESBL and carbapenemase.

				ESBL enzyme			CPE enzyme
		ESBL	CPE	CTX-M-1 gr	SHV-7	SHV-55	OXA-181
Species	n	n (%)	n (%)	n (%)	n (%)	n (%)	n (%)
*Escherichia coli*	31	20 (64.5)	1 (3.2)	20 (100)	0	0	1 (100)
*Klebsiella pneumoniae*	26	20 (76.9)	0	15 (75)	3 (15)	2 (10)	0
*Enterobacter cloacae*	15	8 (53.3)	0	8 (100)	0	0	0
*Other isolates* ^*a*^	10	0	0	0	0	0	0
**Total**	**82**	**48 (58.5)**	**1 (1.2)**	**43 (89.6)**	**3 (6.2)**	**2 (4.2)**	**1 (100)**

^*a*^ Other isolates are *Salmonella* Enteritidis (n = 5), *Morganella morganii* (n = 3), *Proteus mirabilis* (n = 1) and *Leclercia adecarboxylata* (n = 1).

All ESBLs strains carried *bla*_*CTX-M-1 group*_ genes (89.6%) except 5 *K*. *pneumoniae* which carried *bla*_*SHV*_ genes, (3 SHV-7 and 2 SHV-55) (Table 4). The one carbapenemase producing *E*. *coli* also carried a *bla*_*OXA-181*_ gene. It was susceptible to imipenem (MICs of 0.5 mg/L) and meropenem (0.19 mg/L) and intermediately resistant to ertapenem (1 mg/L). It was resistant to all other antibiotics tested except amikacine and tigecycline (MIC of 0.38 mg/L).

**Fig 3:**
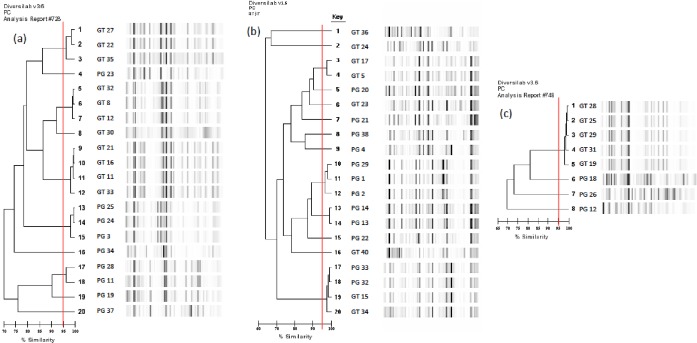
Genetic relatedness (Rep-PCR) between ESBL producing *Enterobacteriaceae* isolated from bacteriemic patients hospitalized in referral centers in Bamako (Mali). (a) ESBL-producing *E*. *coli*, n = 20. (**b**) ESBL-producing *K*. *pneumonia*, n = 20 and (**c**) ESBL-producing *E*. *cloacae*, *n = 8*. PG = Point G University teaching hospital (adults), GT = Gabriel Touré University teaching hospital (pediatric).

ESBL-E relatedness analysis showed that 15/20 (75%) ESBL *E*. *coli*, were distributed in 5 clusters (4 strains in one cluster and 2 in three), the 5 remaining strains (25%) being singletons. Community and hospital-acquired ESBL strains with different susceptibility patterns were mixed in 3 clusters (Fig 3 and S1 Fig). Eleven of the 20 (55%) ESBL *K*. *pneumonia*, were distributed in 4 clusters (2 strains in two clusters, 3 and 4 in the others). *K*. *pneumoniae* that clustered were always from hospital-acquired BSI and shared the same susceptibility patterns (Fig 3 and S2 Fig). One cluster contained strains from both hospitals. *E*. *cloacae* were highly clonal, 5/8 strains (62.5%) clustering together, sharing the same susceptibility pattern and originating from GTUH (Fig 3 and S3 Fig).

## Discussion

Our most important result was that the rate of ESBL nearly reached two third (62.3%) in BSI *Enterobacteriaceae* at two major hospitals in Bamako, the capital of Mali. This is much higher than what has been reported previously in infected hospitalized patients from Africa [11]. This may be because our patients were hospitalized in referral hospitals and had previously stayed in other hospitals where they could have been exposed to antibiotics and may have been in contact with other infected patients. Indeed, previous antibiotics are a major risk factor for ESBL infections [12, 13].

It seems that the increase in ESBL rates in Mali is recent. Ten years ago the prevalence of ESBL-E was only 21.4% at the PGUH hospital [14]. In that study, all clinical strains were included and not only those from blood cultures. It suggests however that ESBL-E incidence may have tripled in the country in ten years. Recently, very high rates of ESBL-E have been reported in clinical samples from hospitalized patients in countries from the region including 52.1% in Nigeria [15], 58% in Burkina Faso [16], 59% in Senegal [17] and 62% in Uganda [18]. Our overall ESBL rates in children were globally similar to those of adults but even higher in hospital-acquired BSI. This corroborates with what has been found in Senegalese [19] and South-African pediatric hospitals [20], emphasizing major risks for the pediatric population in those countries. A recent meta-analysis evoked the recent and dramatic increase of ESBL infections in pediatric patients [13]. The incidence rate of ESBL-E BSI is lower than the one reported in a Senegalese study [19] and could hardly be compared to those of Western countries, taking into account the low number of blood culture drawn in African hospitals.

The mechanism that conferred the ESBL phenotype was overrepresented by CTX-M-1 enzymes (89.6%), as everywhere in Africa and worldwide [21, 22]. Other enzymes, such as SHV, were anecdotal and limited to *K*. *pneumoniae*. Of note, SHV-55 has been reported in Europe (Portugal) and South America (Brazil) but never in Africa [23, 24]. Strikingly, only one of our patients had an *E*. *coli* producing an OXA-181 carbapenemase. CPE have apparently never been described in Mali so far. The patient did not seem to have travelled recently outside of Mali. However, he was previously hospitalized in another hospital in Bamako, suggesting that CPE may already circulate in the community or in Malian health-care structures. Isolating such CPE was indeed not a surprise, since CPE strains have been repeatedly reported in the region and where OXA-48-like enzymes are the most prevalent mechanism conferring resistance [25].

The very high rate of ESBL that we documented here is a warning for the care of patients hospitalized at the referral hospitals of Bamako, where amoxicillin or third generation cephalosporin plus gentamicin, are currently the recommended first line antibiotics for severe infections. Indeed, these regimens will often be suboptimal, thus possibly resulting in inadequate therapy and increased mortality. In Europe, the increased risk of death is well documented in patients with ESBL infections over those with susceptible strains [4]. Also, in teaching hospitals in Dakar, the capital of Senegal, bordering Mali, the length of stay of patients with ESBL infections is prolonged by 4 days, the cost increased by 100 dollars, and the mortality rate multiplied by 5.3 [17]. A change for empiric treatment with carbapenems in patients suspected of ESBL-BSI could reduce mortality but would result in a sharp increase of antibiotic costs. The question is even more complex as the extensive use of carbapenems is followed by an increased risk of CPE colonization and infections [26, 27]. Indeed, another result of our study showed that the rate of CPE BSI was still very low in Mali, in comparison to what is observed in other low and middle-income countries, where carbapenem use has been skyrocketing in recent years [28].

Yet another important result of our study was that that relatedness analysis demonstrated a high probability of cross transmission between patients in Malian hospitals, thus suggesting the necessity for better hygiene measures. Moreover, several clonal ESBL-E strains are also present in both hospitals implying that strains can spread from one hospital to another either because of the personnel working simultaneously in both hospitals, or due to patient transfers. Alcohol-based hand rub (ABHR) are locally produced in Bamako hospitals since 2012 but its actual use has not been monitored. ESBL-E transmission or acquisition rates are poorly documented in Sub-Saharan African hospitals but appear to be high. In a pediatric renutrition center in Niger, the acquisition rate of ESBL-E in children reached 94% after a median length of stay of 8 days [29]. In a pediatric hospital from Gabon, the increase of ESBL-E colonization rate was estimated at 20% for every 48 hours of hospitalization [30]. Both studies highlighted the crucial need for implementing strict control measures to prevent cross transmission as recommended by WHO (www.who.int/gpsc/background/en/).

In conclusion, although we studied only two hospitals that may not be representative of the full healthcare system of the country, our results demonstrate that the rate of ESBL-BSI is very high in hospitalized patients in Bamako, probably as a consequence of previous antibiotic exposure and high cross-transmission rates. This rate appears slightly higher than what has been previously reported in the region, suggesting that the ESBL-E dissemination is not controlled. By contrast, CPE are still rare, suggesting that it is necessary to be cautious when using carbapenems. Therefore, the overall efforts should focus on rational use of antibiotics and better hygiene.

## Supporting information

S1 Fig Genetic relatedness (Rep-PCR) between the 20 ESBL-producing *E*. *coli* from patients hospitalized in referral centers in Bamako (Mali). Pediatric Dpt = Pediatric department, ITD = Infectious and tropical disease unit, ICU = Intensive care unit, HA = Hospitalized acquired BSI, CA = community acquired BSI, Gm = Gentamicin, Sxt = Co-trimoxazole, Fq = Fluoroquinolones, Te = Tetracycline, Fos = Fosfomycin. Adults included were all hospitalized at Point G hospital and children at Gabriel Toure hospital.
(TIFF)

S2 Fig Genetic relatedness (Rep-PCR) between the 20 ESBL-producing *K*. *pneumoniae* from patients hospitalized in referral centers in Bamako (Mali). Pediatric Dpt = Pediatric department, ICU = Intensive care unit, HA = Hospitalized acquired BSI, ND = Not determined, Ak = Amikacin, Gm = Gentamicin, Sxt = Co-trimoxazole, Fq = Fluoroquinolones, Te = Tetracycline. Adults included were all hospitalized at Point G hospital and children at Gabriel Toure hospital.
(TIFF)

S3 Fig Genetic relatedness (Rep-PCR) between the 8 ESBL-producing *E*. *cloacae* from patients hospitalized in referral centers in Bamako (Mali). Pediatric Dpt = Pediatric department, ITD = Infectious and tropical disease unit, ICU = Intensive care unit, HA = Hospitalized acquired BSI, Gm = Gentamicin, Sxt = Co-trimoxazole, Fq = Fluoroquinolones, Te = Tetracycline. Adults included were all hospitalized at Point G hospital and children at Gabriel Toure hospital.
(TIFF)
