# Supplementary figures and images for: Very high prevalence of extended-spectrum beta-lactamase-producing Enterobacteriaceae in bacteriemic patients hospitalized in teaching hospitals in Bamako, Mali
Source: PLoS One. 2017 Feb 28;12(2):e0172652. doi: 10.1371/journal.pone.0172652 (PMC5330466; doi:10.1371/journal.pone.0172652)

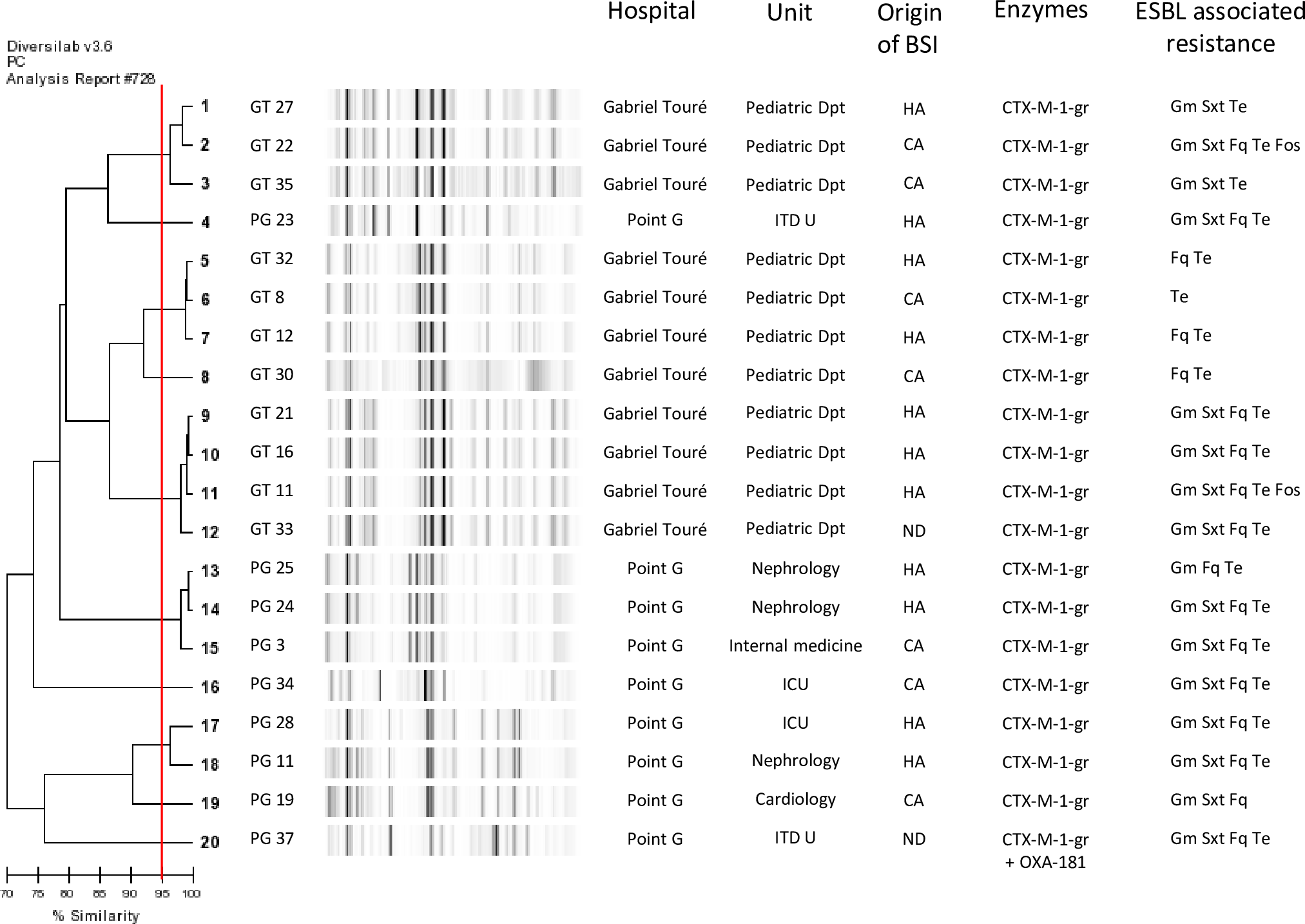

Supplement: S1 Fig — Pediatric Dpt = Pediatric department, ITD = Infectious and tropical disease unit, ICU = Intensive care unit, HA = Hospitalized acquired BSI, CA = community acquired BSI, Gm = Gentamicin, Sxt = Co-trimoxazole, Fq = Fluoroquinolones, Te = Tetracycline, Fos = Fosfomycin. Adults included were all hospitalized at Point G hospital and children at Gabriel Toure hospital. (TIFF) [file pone.0172652.s001.tiff]

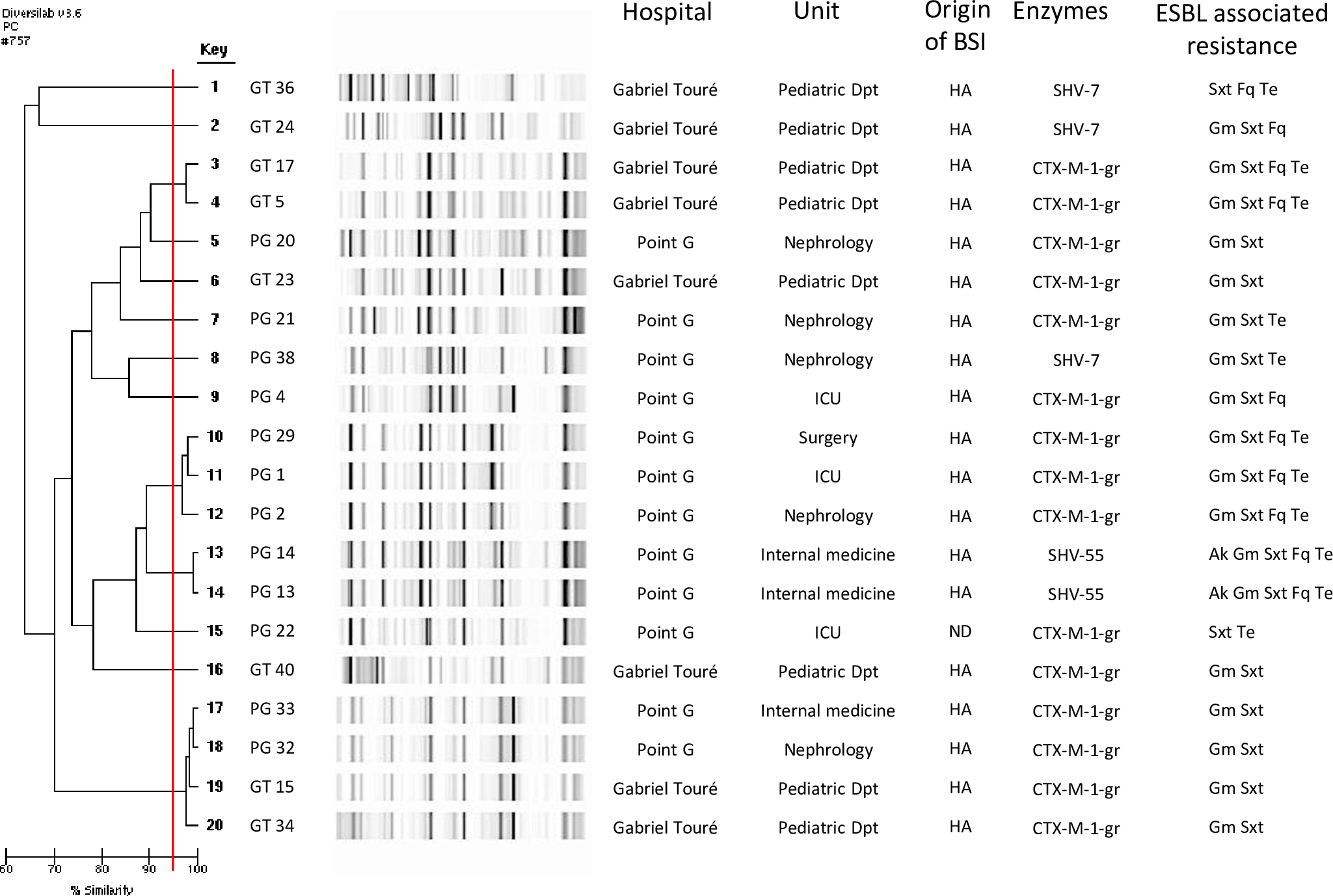

Supplement: S2 Fig — Pediatric Dpt = Pediatric department, ICU = Intensive care unit, HA = Hospitalized acquired BSI, ND = Not determined, Ak = Amikacin, Gm = Gentamicin, Sxt = Co-trimoxazole, Fq = Fluoroquinolones, Te = Tetracycline. Adults included were all hospitalized at Point G hospital and children at Gabriel Toure hospital. (TIFF) [file pone.0172652.s002.tiff]

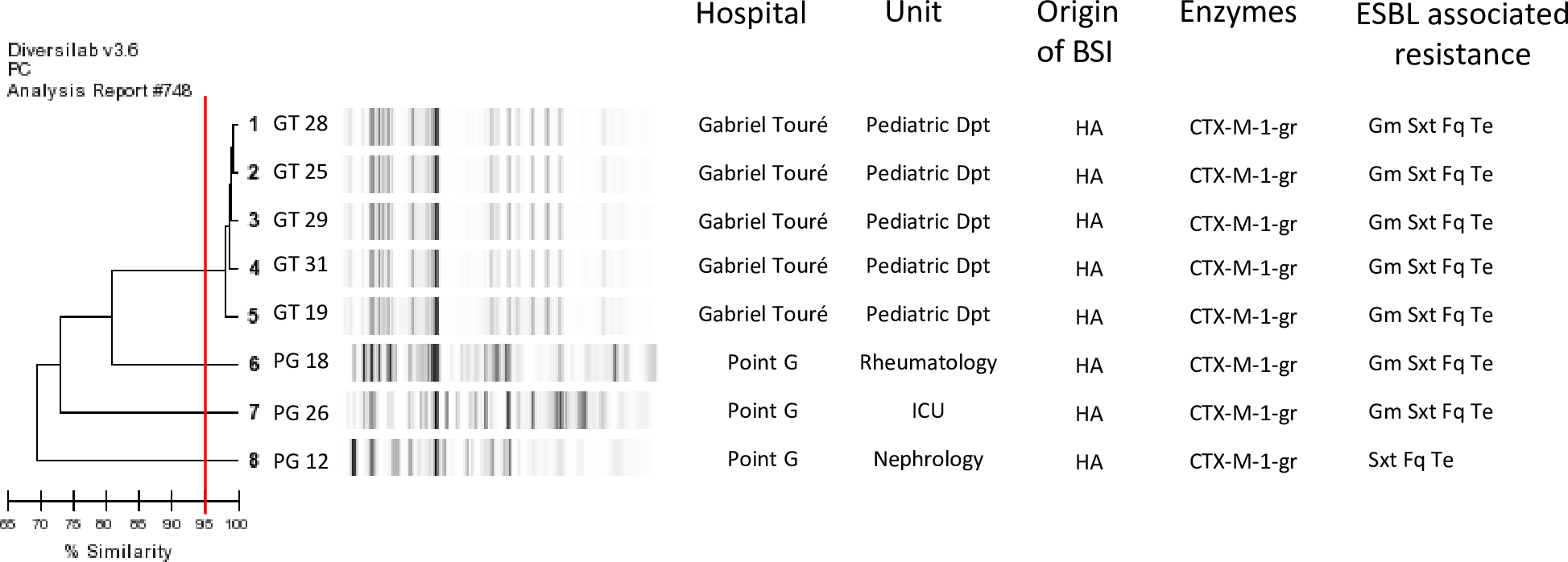

Supplement: S3 Fig — Pediatric Dpt = Pediatric department, ITD = Infectious and tropical disease unit, ICU = Intensive care unit, HA = Hospitalized acquired BSI, Gm = Gentamicin, Sxt = Co-trimoxazole, Fq = Fluoroquinolones, Te = Tetracycline. Adults included were all hospitalized at Point G hospital and children at Gabriel Toure hospital. (TIFF) [file pone.0172652.s003.tiff]
